# Critical WBGT for 4 protective clothing made of fabrics with different Total Heat Loss values

**DOI:** 10.1186/2046-7648-4-S1-A70

**Published:** 2015-09-14

**Authors:** Aitor Coca, Jung-Hyun Kim, Candi Ashley, Thomas Bernard

**Affiliations:** 1 NIOSH/NPPTL, Pittsburgh, PA, USA; 2 University of South Florida, Tampa, FL, USA

## Introduction

Managing heat stress in the workplace while wearing protective clothing (PC) is critical for worker safety and health. There is a variety of PC, such as fire-fighting ensembles, emergency medical clothing, CBRN suits, etc., designed to protect the wearer's body from specific hazards, which have different levels of protection and thus different clothing thermal characteristics (thermal and vapour resistances). This study evaluated the critical wet bulb globe temperature (WBGT_crit_) of PC ensembles with four different total heat loss values determined by a sweating hotplate (SHP) test.

## Methods

Seven healthy male adults participated in this study. Subjects were acclimatized for 5 days prior to the tests of five PC ensembles in random order. The five tests consisted of four PC ensembles with identical design but with different Total Heat Loss (THL) values, and control working clothes. Control is the cotton regular working clothes; PC- A was a prototype with a THL value of 904 W/m^2^; PC-B had a THL value of 700 W.m^-2^; PC-C was another prototype with a THL value of 500 W.m^-2^; and PC-D was a commercially available chemical PE with a THL value of 191 W.m^-2^. Subjects walked on a treadmill at a metabolic work rate of 160 W.m^-2 ^in a climatic chamber that slowly increased the level of heat stress when their core temperature reached a steady-state. WBGT was set at 25.5 (50 %rh) at the start of the session and the ambient temperature was increased 1 degree every 5 minutes after steady-state. The point at which the core temperature began to increase was defined as the inflection point, and the WBGT recorded 5 min before the inflection point was determined as the WBGT_crit _for each PE.

## Results

**Figure 1:**
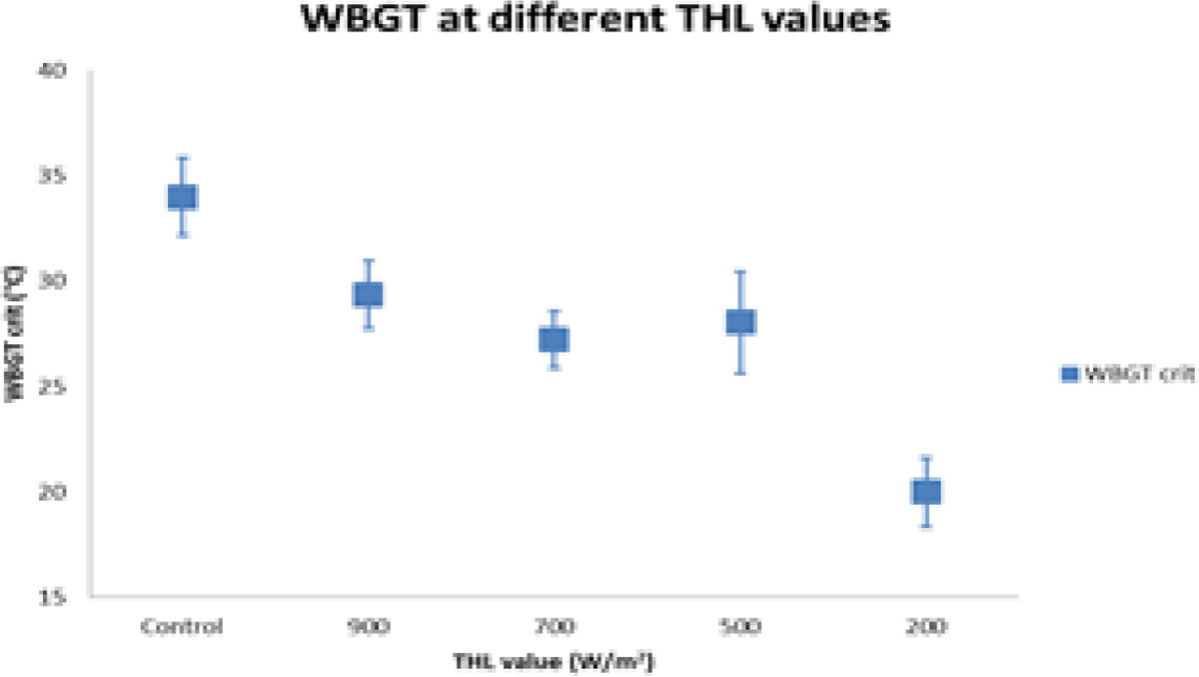
**WBGT_crit _for control work clothing and four PC with different THL values**.

Figure 1 shows the comparison of the different THL values with the WBGT_crit_. For control working clothes, WBGT_crit _is significantly different from any of the PC tested. The PC built with the lowest THL value also has a significantly lower WBGT_crit _than the other PC tested. However, there are no significant differences in WBGT_crit _between PC built from fabrics that are above 500 W/m^2^.

The materials used to manufacture these PC were rated in a range from a very low (PC-D, 191 W.m^-2^) and a very high THL value (PC-A, 904 W.m^-2^). Three of the PC did not show any differences on WBGT_crit_, even with THL differences of about 400 W.m^-2^.

## Conclusion

In summary, the results of this research supports that the SHP-THL value may be effective in distinguishing basic thermal characteristics of the fabrics used for PC; however, the overall effect of fabric THL ratings on PC WBGT_crit _was not linearly related in our study. Moreover, this preliminary data suggest that heat stress caused by PC with different THL values, between 500 and 900 W.m^-2^, may not be physiologically different in terms of WBGT_crit_.

## Disclaimer

The findings and conclusions of this abstract are those of the authors and do not necessarily reflect the views of the National Institute for Occupational Safety and Health.

